# Hierarchical Functionalisation of UiO-66(Zr)-NH_2_ with Cysteine, PEG, and SARS-CoV-2 Spike RBD to Facilitate ACE2 Receptor Targeting in Model Cells

**DOI:** 10.3390/nano16110670

**Published:** 2026-05-26

**Authors:** Veronika Huntošová, Saraa Baddour, Alexandra Migasová, Noémi Bilakovics, Anass Benziane, Michaela Salaková, Zuzana Jurašeková, Tomáš Zelenka, Gabriela Zelenková, Tim Schubert, Florina Zakany, Tamas Kovacs, Arpan Chowdhury, Ľuboš Ambro, Andrea Bodnár, Péter Szűcs, Judit Váradi, Andreas Walter, Erik Sedlák, Miroslav Almáši, György Vámosi

**Affiliations:** 1 Center for Interdisciplinary Biosciences, Technology and Innovation Park, P.J. Šafárik University in Košice, Jesenná 5, SK-041 54 Košice, Slovakia; lubos.ambro@upjs.sk (Ľ.A.); erik.sedlak@upjs.sk (E.S.); 2 Institute of Animal Biochemistry and Genetics, Centre of Biosciences, Slovak Academy of Sciences, Dúbravská cesta 9, SK-840 05 Bratislava, Slovakia; 3 Department of Biophysics and Cell Biology, Faculty of Medicine, Doctoral School of Molecular Medicine, University of Debrecen, Egyetem tér 1, H-4032 Debrecen, Hungary; saraa.baddour@med.unideb.hu (S.B.); bilakovics.noemi@med.unideb.hu (N.B.); benziane.anass@med.unideb.hu (A.B.); florina.zakany@med.unideb.hu (F.Z.); kovacs.tamas@med.unideb.hu (T.K.); chowdhury.arpan@med.unideb.hu (A.C.); bodnar@med.unideb.hu (A.B.); 4 Department of Inorganic Chemistry, Faculty of Science, P. J. Šafárik University in Košice, Moyzesova 11, SK-041 54 Košice, Slovakia; alexandra.migasova@student.upjs.sk; 5 Center for Optical Technologies, Aalen University, Anton-Huber-Straße 21, DE-73430 Aalen, Germany; michaela.salakova@hs-aalen.de (M.S.); tim.schubert@hs-aalen.de (T.S.); andreas.walter@hs-aalen.de (A.W.); 6 Department of Biophysics, Faculty of Science, P. J. Šafárik University in Košice, Jesenná 5, SK-041 54 Košice, Slovakia; zuzana.jurasekova@upjs.sk; 7 Department of Chemistry, Faculty of Science, University of Ostrava, 30. Dubna 22, CZ-702 00 Ostrava, Czech Republic; tomas.zelenka@osu.cz (T.Z.); gabriela.zelenkova@osu.cz (G.Z.); 8 Department of Anatomy, Histology and Embryology, Faculty of Medicine, University of Debrecen, Nagyerdei Körút 98, H-4032 Debrecen, Hungary; szucs.peter@med.unideb.hu; 9 Department of Pharmaceutical Technology, Faculty of Pharmacy, University of Debrecen, Nagyerdei Körút 98, H-4032 Debrecen, Hungary; varadi.judit@pharm.unideb.hu; 10 Department of Biochemistry, Faculty of Science, P. J. Šafárik University in Košice, Moyzesova 11, SK-041 54 Košice, Slovakia

**Keywords:** spike protein, targeted delivery, ACE2 receptors, metal–organic framework, UiO-66(Zr)-NH_2_, cysteine functionalisation

## Abstract

Hierarchical functionalisation of the UiO-66(Zr)-NH_2_ metal–organic framework with cysteine, poly(ethylene glycol) (PEG), and the SARS-CoV-2 spike receptor-binding domain (RBD) was developed to enable receptor-specific interaction with the angiotensin-converting enzyme 2 receptor (ACE2) in model cells. Post-synthetic modification using cysteine and heterobifunctional PEG linkers allowed controlled bioconjugation of SpyTag-labelled RBD via SpyTag/SpyCatcher chemistry, while preserving the crystallinity, microporosity, and intrinsic optical properties of the UiO-66(Zr)-NH_2_ framework. Comprehensive physicochemical characterisation confirmed successful surface functionalisation, tunable aggregation behaviour, and retention of multimodal optical characteristics. Cellular studies in HEK293T and HeLa cells overexpressing EGFP-tagged ACE2 demonstrated enhanced and selective association and uptake of RBD-functionalised nanoparticles compared with non-targeted analogues. Multimodal fluorescence imaging, fluorescence lifetime imaging microscopy, flow-cytometry, and electron microscopy indicated ACE2-dependent endocytic internalisation, with predominant localisation in endosomal and autophagosomal compartments, while both amine- and cysteine-modified formulations exhibited good biocompatibility. Overall, this study establishes a virus-mimetic, ACE2-targeted UiO-66(Zr)-based nanosystem as a proof-of-concept biointerface platform for receptor-specific cellular delivery and imaging, providing a foundation for future MOF-based nanocarriers exploiting ligand–receptor interactions.

## 1. Introduction

Serious infectious diseases and the continual emergence of novel viruses represent a major global health challenge. Effective preparedness at the earliest stages of an outbreak is therefore essential [1]. The 2019 coronavirus pandemic (COVID-19), caused by severe acute respiratory syndrome coronavirus 2 (SARS-CoV-2), has resulted in more than 700 million confirmed infections and over 7 million deaths worldwide [2], with profound consequences for public health, society and the global economy [3]. Such large-scale threats underscore the urgent need for rapid and efficient response strategies, in which prevention, early detection, and targeted therapeutic interventions constitute the core pillars of effective emergency management [4].

Modular nanoparticles as part of nanotechnology-based approaches have shown considerable potential due to their versatility and multifunctional capabilities [5,6]. Such nanoparticles provide therapeutic functions that enable simultaneous therapeutic action and real-time evaluation of treatment efficacy. Among them, inorganic nanoparticles have great potential owing to their inherent antiviral properties, such as disruption of viral structure, generation of reactive oxygen species or local heat-induced viral neutralisation [7,8,9]. In recent years, nanoparticles have been increasingly used for the development of vaccines, targeted delivery systems, diagnostic tools and effective therapies [7,10,11].

Metal–organic frameworks (MOFs) are porous crystalline materials composed of metal ions or clusters coordinated with organic linkers [12]. They can be customised for a wide range of applications and are of considerable interest in medicine due to their high stability, modularity and large porous surface area [13]. The textural properties of MOFs largely determine their potential in the diagnosis and neutralisation of coronavirus SARS-CoV-2 [14]. Recent work from our group demonstrated that MIL-101(Al) can be used for antiviral drug delivery, immobilisation of spike protein, as well as for bioimaging and antibacterial photodynamic treatment [8]. A disadvantage of this system was its large size (several µm), which prevented penetration of the cellular membrane. While these studies primarily addressed cargo loading and antiviral or photodynamic activity, they did not explicitly focus on controlled protein presentation at the nanoparticle–cell interface, which is critical for receptor-specific targeting. The structural design of the nanoparticles builds on our previous work with histidine-modified MOFs [15].

UiO-66(Zr)-NH_2_ (UiO = University of Oslo) is a zirconium-based metal–organic framework consisting of [Zr_6_O_4_(OH)_4_(COO)_12_] inorganic clusters interconnected by 2-aminoterephthalate organic linkers, forming a highly stable, microporous three-dimensional network. UiO-66(Zr)-NH_2_ is a versatile MOF material [16] with exceptionally high thermal and chemical stability, which, together with its large surface area and tuneable pore structure, makes it attractive for targeted delivery applications [17,18,19]. UiO-66(Zr)-NH_2_ nanoparticles have been used against influenza A virus in mice, where they activated the RIG-I-like receptor signalling pathway [10]. Through surface modification with silver nanoparticles, UiO-66(Zr)-NH_2_ can also be applied in water disinfection [20]. Owing to their tunable properties and small size, these nanoparticles can further serve as a platform for pulmonary drug delivery [21]. UiO-66(Zr)-NH_2_ is biocompatible and therefore suitable for the administration of anti-inflammatory drugs to promote wound healing [22]. Moreover, surface modification with poly(ethylene glycol) chains improves phosphate stability and enables *pH*-dependent cargo release in cancer target tissues [23]. Recently, the potential of MOFs against SARS-CoV-2 has also been investigated, including UiO-66(Zr)-based systems [24].

The biocompatibility and targeting efficiency of nanoparticles can be enhanced by surface functionalisation with active groups such as amino, carboxyl, and thiol moieties [25,26,27]. After successful histidine modification, this work focused on cysteine (Cys) functionalisation. In contrast to histidine, Cys introduces redox-sensitive thiol functionality, offering additional control over protein conjugation chemistry and nanoparticle aggregation behaviour at the biointerface. Cys modification offers significant potential for the development of responsive delivery systems due to the formation of disulfide bonds, which can serve as crosslinks in response to the local microenvironment [28]. Moreover, Cys residues are fundamental for proper protein folding, stabilisation of tertiary structure, and coordination of metal ions [29,30]. These characteristics are particularly relevant for nanoparticle functionalisation with protein, as they facilitate specific interactions such as receptor-targeted binding.

Angiotensin-converting enzyme 2 (ACE2) has been identified as the primary cellular receptor for the SARS-CoV-2 virus and the main entry point for viral infection [31]. Consequently, strategies aimed at blocking the interaction between SARS-CoV-2 and the ACE2 receptor (denoted as ACE2r in nomenclature) represent a highly attractive therapeutic approach for controlling disease progression [32].

Despite advances in antiviral strategies, approaches enabling receptor-specific targeting remain limited, particularly those adaptable to emerging variants. The approach presented in this study represents a promising alternative, as it may improve the selectivity of antiviral agents through targeted delivery and could retain effectiveness against multiple variants via ACE2 targeting.

**Figure 1:**
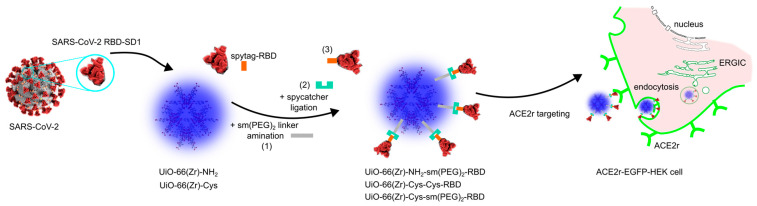
Schematic representation of experimental design.

Here, UiO-66(Zr)-NH_2_ nanoparticles were prepared for ACE2 targeting through hierarchical surface functionalisation with cysteine, PEG linkers, and the SARS-CoV-2 RBD (Figure 1). Cellular uptake and intracellular localisation were evaluated in model cell lines overexpressing ACE2 receptors.

## 2. Materials and Methods

The methods were organised to progressively link surface chemistry with biological performance: nanoparticle synthesis and functionalisation were followed by physicochemical validation and, subsequently, receptor-targeted cellular studies.

### 2.1. Synthesis of UiO-66(Zr)-NH_2_ and the Modification of Surface by Cysteine

All chemicals used for synthesis were purchased from Sigma-Aldrich (Sigma-Aldrich, Merck KGaA, Darmstadt, Germany) in the highest purities and were used without any further purification.

The base material UiO-66(Zr)-NH_2_ was synthesised and subsequently activated according to the procedure described in the literature [15,33], with no further modifications.

**Figure 2:**
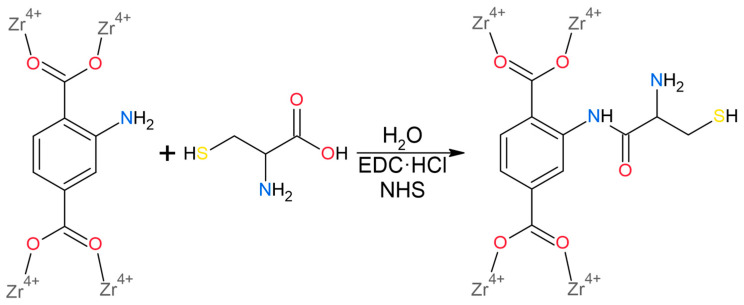
Schematic representation of cysteine grafting onto the 2-aminoterephtalate linker of the UiO-66(Zr)-NH_2_ framework. The 2-aminoterephthalate linker constitutes the organic component of the UiO-66(Zr)-NH_2_ framework and provides reactive -NH_2_ groups for post-synthetic modification.

The post-synthetic surface modification was carried out via amide bond formation between the free –NH_2_ groups of the 2-aminoterephthalate linker within the UiO-66(Zr)-NH_2_ framework and the carboxyl group of cysteine. The reaction scheme illustrating this functionalisation is shown in Figure 2. L-cysteine (denoted as Cys) (51.8 mg, 0.43 mmol, 1.0 eq.) was dissolved in water, followed by the addition of 1-ethyl-3-(3-dimethylaminopropyl)carbodiimide hydrochloride (EDC·HCl) (136.6 mg, 0.71 mmol, 2.5 eq.) and *N*-hydroxysuccinimide (NHS) (82 mg, 0.71 mmol, 2.5 eq.). The reaction mixture was cooled in an ice bath, after which UiO-66(Zr)-NH_2_ (500 mg, 0.285 mmol) was added and the suspension was stirred overnight. The resulting solid UiO-66(Zr)-NH_2_-Cys (denoted as UiO-66(Zr)-Cys) was collected by filtration, purified by Soxhlet extraction using methanol as the solvent, and subsequently thermally activated at 80 °C. The yield of the dried UiO-66(Zr)-Cys product was 493 mg (92% based on UiO-66-NH_2_).

### 2.2. Characterisation

Fourier-transform infrared (FTIR) spectra were recorded using a Nicolet Avatar 6700 spectrometer (Thermo Fisher Scientific, Waltham, MA, USA). The measurements were performed in transmission mode using KBr pellets prepared at a mass ratio of 1:100 (sample:KBr). Spectra were collected in the 4000–400 cm^−1^ range with a spectral resolution of 4 cm^−1^ and 64 scans per spectrum. To improve the detection of weak thiol vibrations, an additional spectral window from 2700 to 2400 cm^−1^ was acquired with 128 scans and a resolution of 1 cm^−1^.

Powder X-ray diffraction (PXRD) patterns were recorded using a Bruker D2 Phaser diffractometer (Bruker, Berlin, Germany) operated in Bragg–Brentano geometry and equipped with a Cu K*α* radiation source (*λ* = 1.54056 Å) at 45 kV and 40 mA. The instrument was fitted with a LynxEye detector (Bruker, Berlin, Germany), and the measurements were performed using a fixed divergence slit. Diffraction data were collected over a 2*θ* range of 5–50° with a step size of 0.1° and a scan speed of 3° min^−1^. The obtained patterns were processed and evaluated using the DIFFRAC.EVA software package version 5.1 (Bruker).

The thermal stability of the prepared materials was evaluated by thermogravimetric analysis (TGA) using a Setsys Evolution instrument (Setaram, Caluire, France). Approximately 20 mg of each sample was placed into *α*-Al_2_O_3_ crucibles, and the measurements were performed under an air atmosphere consisting of 20% O_2_ and 80% Ar with a total gas flow rate of 40 mL min^−1^. The samples were heated from 25 to 800 °C at a rate of 10 °C min^−1^. Because the samples contained different amounts of physisorbed and occluded solvent molecules within their frameworks, all thermoanalytical curves were normalised at 200 °C to eliminate the contribution of physically bound volatiles [34,35]. The mass loss associated with solvent removal was therefore redistributed between the mass loss attributable to the decomposition of the organic linker components and the final residual mass corresponding to ZrO_2_.

Elemental analysis was performed to determine the C, H, N and S contents (wt.%) of the prepared materials using a Vario MICRO CHNS elemental analyser (Elementar Analysensysteme GmbH, Langenselbold, Germany). Approximately 2 mg of each activated sample was used for the measurement.

The textural properties (BET area (*S_BET_*), pore volume (*V_p_*) and the most frequent pore diameter (*d*)) were determined using argon adsorption/desorption experiments measured at −186 °C, while the gas purity was 99.999%. Adsorption measurements were carried out on an Autosorb iQ-XR static adsorption system from Quantachrome Instruments (Boynton Beach, FL, USA), as described elsewhere [36,37,38]. The isothermal conditions of −186 °C were maintained with a CryoSync device from Quantachrome Instruments in combination with a liquid nitrogen bath. The adsorption/desorption measurements were performed at the relative pressure (*p*/*p_0_*) from 2.10^−4^ to 0.995. The isotherms were analysed using the ASiQwin software package version 5.0 from Quantachrome Instruments to determine the required textural properties. The *S_BET_* was determined based on Rouquerol’s consistency criteria [39,40]. The *V_p_* represents the pore volume, corresponding to the total volume of pores smaller than 77 nm. The most frequent pore diameter, *d*, was determined by fitting the experimental adsorption isotherm data using the NLDFT kernel, assuming cylindrical silica pores.

### 2.3. FIBSEM

The dimensions of the prepared particles were measured with a Focused Ion Beam Scanning Electron Microscope (FIBSEM), Crossbeam 540 by Zeiss (Oberkochen, Germany). UiO-66(Zr)-NH_2_ and UiO-66(Zr)-Cys nanoparticles were suspended in demineralised water and mixed well using a pipette. Subsequently, they were distributed on the surface of EM pin sample plates and allowed to dry. Samples were covered with a 2 nm gold layer via sputter deposition to enhance conductivity. Data were collected at 1.5 kV, 300 pA, with a dwell time of 50 ns, and a pixel dimension of 2.23 nm (x/y).

### 2.4. Raman Spectroscopy

Raman spectra of UiO-66(Zr), UiO-66(Zr)-NH_2_ and UiO-66(Zr)-Cys particles in powder (solid state) were acquired using a Renishaw inVia Raman microspectrometer (Wotton-under-Edge, Gloucestershire, UK) with an excitation laser wavelength of 785 nm. The spectra were recorded over the spectral range of 100–4000 cm^−1^ to enable the capture of fluorescence background in addition to the Raman signal. The signal was initially calibrated using the 520 cm^−1^ line of a silicon wafer. All measurements were carried out in micro mode utilising a 20× objective lens. The laser power at the sample was adjusted according to the specific characteristics of each sample/measurement to achieve an optimal signal-to-noise ratio while avoiding sample damage and did not exceed 2 mW. For each sample, five independent measurements were obtained, and the spectra presented herein represent the average of them. Data processing and spectral visualisation were carried out using OriginLab Pro 7.5 (OriginLab, Northampton, MA, USA) data analysis and graphing software. For direct comparison, the Raman spectra were normalised to the band at 1616–1625 cm^−1^.

### 2.5. Fluorescence Spectroscopy

Fluorescence spectra of 10 µL of 2 mg/mL UiO-66(Zr)-NH_2_ and UiO-66(Zr)-Cys in 2 mL PBS solution (*pH* = 7.4) were excited at 280 nm and 405 nm. The fluorescence emission spectra were detected in the spectral ranges of 320–530 nm and 420–530 nm using a JASCO spectrofluorometer (FP-8550, Easton, MD, USA).

### 2.6. Modification of Nanoparticles with SARS-CoV-2 RBD-SD1 Protein

Functionalisation of nanoparticles was performed as follows: 100 µL of 2 mg/mL UiO-66(Zr)-NH_2_ or 200 µL of 2 mg/mL UiO-66(Zr)-Cys suspensions in PBS were mixed with 40 µL of 25 mM solution of heterobifunctional PEG crosslinker solution (sm(PEG)_2_) (Thermo Fisher Scientific, Pierce Biotechnology, Rockford, IL, USA; Cat. No. A35397). sm(PEG)_2_ is a hetero-bifunctional crosslinker with terminal *N*-hydroxysuccinimide (NHS) ester and maleimide groups, which enables covalent conjugation to NH_2_ groups and thiol (-SH) groups (of the Cys modification) on the nanoparticles, respectively, while leaving the other functional group available for the next coupling step. The mixtures were gently shaken during incubation at room temperature for 1 h. The nanoparticles were then washed with PBS and resuspended in 100 µL of PBS.

UiO-66(Zr)-NH_2_-sm(PEG)_2_ nanoparticles were mixed with 10 µL of 1.8 mg/mL SpyCatcher003 (Kerafast, Inc., Boston, MA, USA; Cat. No. EOX004) carrying a free SH group due to a mutation (S49C), which enabled covalent bonding to the maleimide on the crosslinker.

Alternatively, UiO-66(Zr)-Cys-sm(PEG)_2_ nanoparticles were incubated with 10 µL of SpyCatcher2 (Bio-Rad Laboratories, Hercules, CA, USA; Cat. No. TZC001), which was supplied at ~250 µM (~3.9 mg/mL) in solution according to the manufacturer’s datasheet.Both reactions were carried out at room temperature for 2 h. The nanoparticles were then washed with PBS and resuspended in 100 µL of PBS.

The SpyTag-RBD (SARS-CoV-2 recombinant RBD-SD1, Friedrich-Loeffler-Institut, Greifswald-Insel Riems, Germany) protein (hereinafter RBD) was labelled with AlexaFluor 488 and AlexaFluor 647 (ThermoFisher Scientific, Waltham, MA, USA) for 50 min (hereinafter RBD-A488 and RBD-A647) and separated using Sephadex G50 (ThermoFisher Scientific). SpyTag can bind covalently to SpyCatcher to create a SpyTag/SpyCatcher system, a modular protein assembly tool.

SpyCatcher-modified nanoparticles were incubated with 20 µL of RBD/RBD-A488 (0.4 mg/mL). UiO-66(Zr)-Cys-sm(PEG)_2_ nanoparticles were incubated with 70 µL of RBD-A647 (0.114 mg/mL). The mixtures were incubated overnight at 4 °C. Following conjugation, potential aggregates were removed by centrifugation at 13,000 rpm for 30 min. The final product was then resuspended in 300 µL of PBS. Alternatively, modified nanoparticles were coupled with non-labelled RBD protein using a similar procedure.

UiO-66(Zr)-Cys nanoparticles were also modified directly through Cys-Cys bonds. A mixture of 10 µL of SpyCatcher003 (S49C) and 200 µL of UiO-66(Zr)-Cys nanoparticles was incubated overnight at 4 °C. The nanoparticles were then washed and resuspended in 100 µL of PBS. Subsequently, 20 µL of RBD-A488 (0.4 mg/mL) was added to the nanoparticle solution and incubated overnight at 4 °C. The nanoparticles were then washed with PBS and resuspended in 300 µL of PBS.

To evaluate the binding efficiency of fluorescently labelled RBD proteins to the nanoparticles, parallel control reactions were prepared and analysed for the amount of unbound RBD-A488 or RBD-A647 remaining in the supernatant after conjugation. Following incubation, nanoparticles were sedimented by low-speed centrifugation and the supernatants were collected for further analysis. Proteins present in the supernatants were precipitated by adding ice-cold trichloroacetic acid (TCA) to a final concentration of 10% (*w*/*v*) and incubated on ice for 1 h. Samples were then centrifuged at 18,000× *g* for 30 min at 4 °C. The resulting pellets were washed with ice-cold acetone and centrifuged again at 18,000× *g* for 5 min at 4 °C. After air drying, protein pellets were resuspended in 20 µL of distilled water. Half of each prepared sample (10 µL) was then analysed by sodium dodecyl sulphate polyacrylamide gel electrophoresis.

### 2.7. Dynamic Light Scattering Measurements of Modified Nanoparticle Parameters

The particle size of nanoparticle-agglomerates was measured using a Zetasizer Nano ZSP (Malvern Panalytical Ltd., Malvern, UK). The test parameters were as follows: three measurements were performed, each consisting of 11 runs; the temperature was 25 °C, and measurements were conducted in a high-concentration zeta cell.

### 2.8. Cell Culture Preparation and Transfection

The HEK293T human embryonic kidney cell line (ATCC, Manassas, VA, USA) and HeLa human cervical carcinoma cells (Cell Lines Services, Eppelheim, Germany) were grown in phenol red–containing Dulbecco’s modified Eagle’s medium supplemented with 10% foetal bovine serum (Sigma-Aldrich, St. Louis, MO, USA), GlutaMAX (L-alanyl-L-glutamine, Fisher Scientific), and 50 mg/L gentamycin (KARA, Novo Mesto, Slovenia). For the microscopy experiments, HEK293T cells were seeded in 8-well chambered coverslips (ibidi, Munich, Germany) and maintained in phenol red-free medium. Twenty-four hours after seeding, the cells reached 50–60% confluence and were transiently transfected with 75–80 ng of ACE2-EGFP plasmid (pcDNA3.1-ACE2-GFP, a gift from Utpal Pajvani (Addgene plasmid #154962; http://n2t.net/addgene:154962 (accessed on 17 November 2025); RRID:Addgene_154962)) (REF: PMID: 33963249) using the FuGENE^®^ HD transfection reagent (Promega, MA, USA), as recommended by the manufacturer. The cells (referred to as ACE2r-EGFP-HEK) were used for microscopy within 24 h after transient transfection.

### 2.9. Confocal Fluorescence Imaging of Nanoparticles and Time-Resolved Fluorescence Lifetime Imaging of ACE2r-EGFP-HEK Cells

UiO-66(Zr)-NH_2_ nanoparticles at a final concentration of 0.001–0.07 mg/mL were incubated with HEK293T or ACE2r-EGFP-HEK cells for 24 h prior to imaging. RBD-modified UiO-66(Zr)-NH_2_-sm(PEG)_2_ nanoparticles were incubated with HEK293T and ACE2r-EGFP-HEK cells for 5 h before imaging. RBD-A488-modified nanoparticles were incubated with HEK293T and ACE2r-EGFP-HEK cells for 2 h prior to imaging and fluorescence lifetime imaging (FLIM) analysis. Confocal images were acquired using an A1 confocal fluorescence microscope equipped with a Plan-Apochromat 60×, NA 1.27 water immersion objective (Nikon, Tokyo, Japan). The confocal microscope was also equipped with a time-correlated single photon counting upgrade kit (PicoQuant, Berlin, Germany), which was used to detect fluorescence lifetimes. Before measurements preview images were taken using the 405 nm and 485 nm continuous wave lasers and detected through 430/475, 500/550 bandpass filters to visualise the fluorescent proteins. Images of 1024 × 1024 pixels were recorded with a zoom of 3 (pixel size: 69 nm). For lifetime measurements, EGFP and Alexa Fluor 488 were excited with a 485 nm pulsed laser at a 20 kHz repetition rate. Fluorescence emissions were detected by a PMA Hybrid 40 photon-counting photomultiplier (Picoquant) through a 520/35 nm emission filter. Lifetime data were collected for 2 min at 37 °C. Between 7 and 13 cells were measured from each sample. To analyse the fluorescence decay curves, SymphoTime 64 software (Picoquant, Berlin, Germany) was used. Prior to fluorescence lifetime determination, images were intensity thresholded to exclude background pixels, and the cell of interest was selected by applying a freehand-drawn region of interest. Fluorescence intensity decay curves for the selected regions of interest were fitted with a multiexponential reconvolution model with two lifetime components:

$$y\left(t\right)=\sum_{i=1}^{2}IRF\;⨂{|}_{{Bkgr}_{IRF}|{Shift}_{IRF}}\;A\left[i\right]\;exp\left(-\frac{t}{\tau \left[i\right]}\right)+{Bkgr}_{Dec}$$

where *t* is time, and *A[i]* and *τ[i]* are the amplitude and exponential decay time of the *ith* component, *Bkgr_IRF_* is the background correction for the instrument response function (IRF), *Shift_IRF_* is the correction for temporal IRF displacement, *Bkgr_Dec_* is the background correction and *τ_Av,amp_* amplitude-weighted average lifetime.

Confocal fluorescence imaging of HeLa cells incubated for 5 h with the nanoparticles was performed to assess the colocalisation of blue-fluorescent nanoparticles, ACE2r-EGFP, and RBD-A647 spike protein. Imaging was conducted using a Zeiss LSM 880 laser scanning microscope (Carl Zeiss, Jena, Germany) equipped with a 40×, NA 1.2 water immersion objective. The nanoparticles were excited with a 405 nm diode laser, and their blue fluorescence emission was collected between 415 and 490 nm; EGFP was excited using the 488 nm line of an Argon-ion laser, with emission detected between 500 and 534 nm; Alexa Fluor 647 was excited by a 633 nm HeNe laser, and its emission was detected between 638 and 756 nm. Images of 2048 × 2048 pixels were recorded with a zoom of 1.42× (pixel size: 72 nm). The experiment was performed at room temperature (23 °C).

### 2.10. Flow Cytometry Analysis of Nanoparticle Uptake by HEK293T and ACE2r-EGFP-HEK Cells

UiO-66(Zr)-NH_2_ nanoparticles at a final concentration of 0.001–0.07 mg/mL were incubated with HEK293T and ACE2r-EGFP-HEK cells for 24 h prior to flow cytometry analysis. RBD-modified UiO-66(Zr)-NH_2_-sm(PEG)_2_ nanoparticles were incubated with HEK293T/HeLa and ACE2r-EGFP-HEK/HeLa cells for 5 h before to flow cytometry analysis. RBD-A488/A647-modified nanoparticles were incubated with cells for 2 and 5 h prior to flow cytometry analysis. The cell-associated signal, including the bound but not internalised nanoparticles, was detected using a NovoCyte 3000 RYB flow cytometer (Agilent, Santa Clara, CA, USA). A 405 nm laser was used to excite UiO-66(Zr)-NH_2_ and UiO-66(Zr)-Cys nanoparticles, a 488 nm laser to excite Alexa Fluor 488 and EGFP, and a 640 nm laser to excite Alexa Fluor 647; fluorescence emissions were detected through 450/50, 530/30, and 660/20 filters, respectively. Forward and side scatter (FSC and SSC) signals of the 488 nm line were also detected. The analysis was performed using FCS Express 6 software (De Novo Software, Pasadena, CA, USA).

### 2.11. Electron Microscopy of Nanoparticle Distribution in ACE2r-EGFP-HEK Cells

Cells were seeded in a 16-well plate on ACLAR^®^33C embedding film (7.mil thickness) (Electron Microscopy Sciences, Hatfield, PA, USA), transfected with ACE2r-EGFP after 24 h, and exposed to nanoparticles for 3 h. After incubation, the cells were fixed with a mixture of 2.5% glutaraldehyde and 2% paraformaldehyde for 2 h at room temperature, then overnight in the fridge (8 °C). The cells were washed with cacodylate buffer (0.1 M) and postfixed for 1 h in 1% osmium tetroxide (OsO4) in cacodylate buffer (0.1 M). This step was followed by three thorough washes in cacodylate buffer (15 min each). Cells were dehydrated in a graded series of ethanol (50%, 70%, 96%, and absolute ethanol), treated in a graded propylene oxide-araldite mixture, and finally embedded in araldite (adhesive). The polymerisation of araldite to form specimen blocks was accomplished in an oven at 56 °C for 48 h. The specimen blocks were trimmed with a Leica Ultracut UCT ultramicrotome (Leica Microsystems GmbH, Wetzlar, Germany). The sections were stained in alcoholic uranyl acetate and lead citrate before examining the grids in a JEOL 1400 Flash transmission electron microscope (JEOL, Tokyo, Japan).

### 2.12. Metabolic Activity of HEK293T Cells in the Presence of UiO-66(Zr)-NH_2_ Nanoparticles

The MTT assay was performed as described previously [15]. Cells seeded in 96-well plates were treated with nanoparticles at varying concentrations (0.002–0.070 mg/mL) for 48 h, after which cell viability was assessed. The cell culture medium was aspirated and replaced by 80 µL of MTT solution (3-(4,5-dimethylthiazol-2-yl)-2,5-diphenyltetrazolium bromide) in each well for 4 h at 37 °C. The solution was then discarded, and formazan crystals were dissolved in 80 µL DMSO and incubated overnight with constant shaking. The optical density was measured using an EnVision 2000 (PerkinElmer, Shelton, CT, USA) microplate reader at 590 nm. Formazan production (%) was evaluated in the studied samples relative to the untreated control.

## 3. Results

### 3.1. Characterisation of UiO-66(Zr)-NH_2_ and UiO-66(Zr)-Cys Nanoparticles and Their Functionalisation with RBD

**Figure 3:**
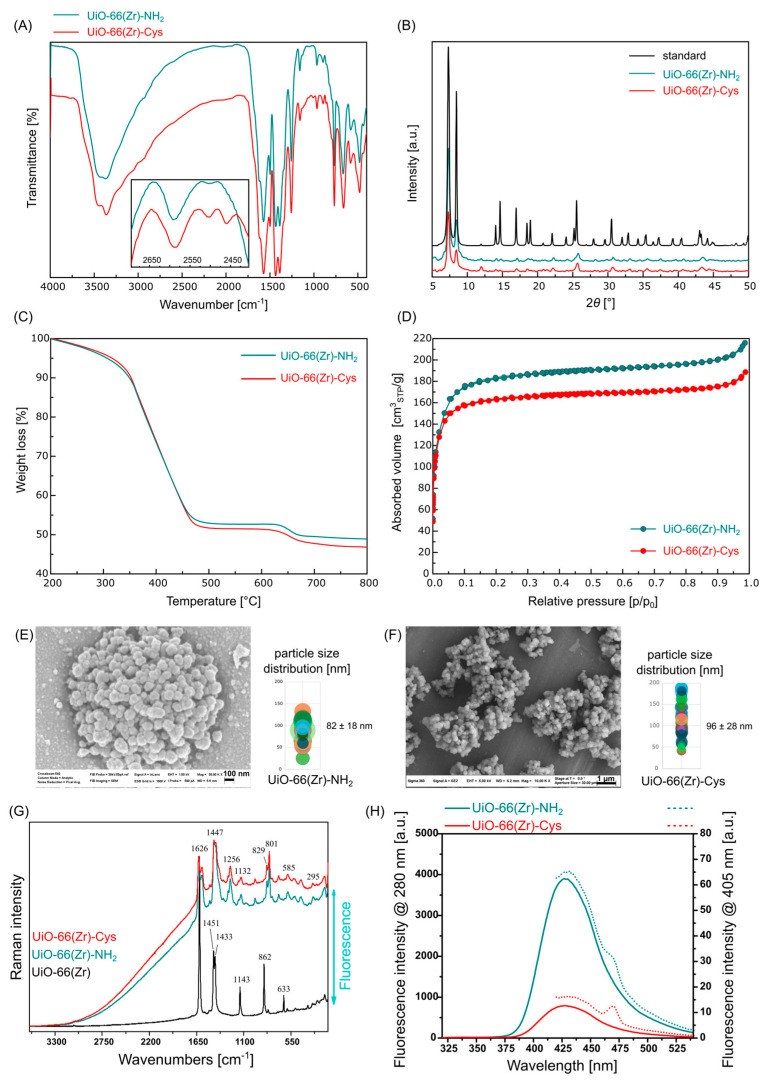
Characterisation of the prepared compounds: (**A**) FTIR spectra for the unmodified and modified materials, (insert) FTIR spectra in the range from 2700 cm^−1^ to 2400 cm^−1^, (**B**) simulated PXRD pattern of UiO-66(Zr)-NH_2_ calculated from the single-crystal X-ray data served as reference (black curve) and measured pristine UiO-66(Zr)-NH_2_ (cyan curve) and surface-modified UiO-66(Zr)-Cys material (red curve), (**C**) the thermogravimetric curves for UiO-66(Zr)-NH_2_ and UiO-66(Zr)-Cys, and (**D**) argon adsorption/desorption isotherms of the prepared materials measured at −186 °C. (**E**) FIBSEM images of nanoparticle agglomerates composed of UiO-66(Zr)-NH_2_ and (**F**) UiO-66(Zr)-Cys. The corresponding particle size distribution within the agglomerates is shown in graphs, where circle diameters indicate the relative frequency of particles of a given size. (**G**) Raman spectroscopy of UiO-66(Zr) (black line), UiO-66(Zr)-NH_2_ (cyan line) and UiO-66(Zr)-Cys (red line) nanoparticles. (**H**) Fluorescence spectra of 0.01 mg/mL nanoparticles dispersed in PBS at *pH* = 7.4. Emission was detected after 280 nm (solid lines) and 405 nm (dotted lines) excitation.

The structural integrity of pristine UiO-66(Zr)-NH_2_ and its Cys-modified analogue was first evaluated by infrared spectroscopy (FTIR) (Figure 3A). The spectra clearly reflect the presence of the framework’s structural building blocks, namely the 2-aminoterephthalate linker and the Zr_6_O_4_(OH)_4_(COO)_12_ cluster, as well as the successful incorporation of Cys (Figure 2). For pristine UiO-66(Zr)-NH_2_, two characteristic amine stretching vibrations, *ν*(NH_2_), appear at 3425 and 3372 cm^−1^. In the Cys-modified material, a marked reduction in amine stretching intensity was observed, consistent with partial consumption of –NH_2_ groups during amide bond formation. Bands corresponding to aromatic C–H stretching vibrations *ν*(CH) are observed in the 2925–2800 cm^−1^ region. In UiO-66(Zr)-Cys, additional bands in the 3200–2925 cm^−1^ interval emerge, attributable to *ν*(CH_2_) stretching of the cysteine methylene groups. The deformation vibration *δ*(N–H) appears at 1573 cm^−1^, while a sharp band at 1499 cm^−1^ is assigned to the *ν*(C=C) absorption bands of the aromatic scaffold. Both materials display strong carboxylate vibrations at 1434 and 1389 cm^−1^, corresponding to asymmetric *ν*(COO^−^)_as_ and symmetric *ν*(COO^−^)_s_ stretching of the coordinated 2-aminoterephthalate linker. The fingerprint region (1258–894 cm^−1^) contains multiple bands associated with *ν*(C–C), *ν*(C–N) stretching, and *δ*(C–H) deformation, reflecting the well-preserved structure of the organic framework. In the 767–582 cm^−1^ region, several sharp bands corresponding to *δ*(Zr–O–Zr) vibrations confirm the presence of the characteristic Zr_6_O_4_(OH)_4_(COO)_12_ secondary building unit [15,41]. Because the thiol (–SH) group exhibits inherently weak absorption in the 2600–2250 cm^−1^ region, an expanded spectral window (2700–2400 cm^−1^) was recorded for more sensitive detection (inset in Figure 3A). In UiO-66(Zr)-Cys, two distinct bands at 2504 and 2460 cm^−1^ were observed, which are assigned to *ν*(S–H) stretching vibrations and provide direct evidence for the presence of Cys residues on the MOF surface [42].

PXRD patterns (Figure 3B) revealed that both pristine and Cys-modified UiO-66(Zr)-NH_2_ retained the characteristic reflections of the UiO-66 framework [43]. The simulated PXRD pattern serves as a reference for structural validation, while comparison between UiO-66(Zr)-NH_2_ and UiO-66(Zr)-Cys highlights the effect of surface modification. The positions of all diffraction peaks remain unchanged after post-synthetic modification, demonstrating that the framework retains its structural integrity and crystallinity throughout the functionalisation procedure. The measured reflections at 2*θ* = 7.33, 8.46, 11.99, 14.12, 14.73, 17.06, 18.61, 19.12 and 22.22° correspond to the (111), (200), (220), (311), (222), (400), (331), (420) and (333) crystallographic planes, respectively, confirming the face-centred cubic structure of UiO-66(Zr)-NH_2_ with space group Fm-3m. No additional peaks attributable to crystalline impurities or degradation products (e.g., ZrO_2_ or linker-derived phases) were detected, further verifying the chemical stability of the MOF during Cys grafting. The lattice parameter *a*, calculated from Bragg’s equation and the assigned *hkl* indices, was 20.70 Å for both samples, closely agreeing with the reference cell lattice parameter (*a* = 20.75 Å) derived from a single-crystal X-ray structure. The overall peak widths and relative intensities remain essentially unchanged, indicating that the functionalisation does not significantly affect the crystallite size or induce measurable framework distortion. These results collectively confirm that Cys is grafted onto the MOF external surface without disrupting the long-range order of the UiO-66(Zr)-NH_2_ architecture.

Having confirmed preservation of the crystalline framework after Cys grafting, we next quantified the extent of surface functionalisation using complementary thermal and elemental analyses.

The thermal behaviour of the materials was analysed to quantify the amount of Cys incorporated into UiO-66(Zr)-Cys, and the resulting thermogravimetric curves are shown in Figure 3C. Both samples exhibit a major mass-loss in the 200–500 °C region, corresponding to the decomposition of the organic moiety, the 2-aminoterephthalate linker and the grafted Cys molecules. At higher temperatures (500–800 °C), the decomposition is completed, and the remaining residue corresponds to crystalline ZrO_2_, which is consistent with the expected thermal decomposition pathway of UiO-66(Zr) materials. By comparing the total mass losses and residual masses of UiO-66(Zr)-NH_2_ and UiO-66(Zr)-Cys, the amount of Cys bound to the framework was calculated to be 22.6 mg per gram of MOF, corresponding to 0.217 mmol g^−1^. Considering that the parent UiO-66(Zr)-NH_2_ contains 3.420 mmol g^−1^ of accessible amine groups, the grafting efficiency corresponds to approximately 6.35% functionalisation of the available –NH_2_ sites. This degree of substitution indicates that, on average, approximately one out of sixteen amine groups is modified with Cys, while the majority of –NH_2_ sites remain unreacted and available for subsequent conjugation with SM(PEG)_2_–RBD–A488. Importantly, only a low density of surface-bound Cys groups is required to enable efficient coupling of the bulky Cys–RBD–A488 biomolecule. Therefore, the observed low degree of Cys functionalisation in UiO-66(Zr)-Cys is advantageous and sufficient for the intended bioconjugation strategy.

Elemental analysis was employed to further confirm the presence and quantify the amount of grafted cysteine. Activated samples, free of residual solvents, were subjected to CHNS elemental analysis. For UiO-66-NH_2_, the experimentally determined values (C 32.73%, H 2.18%, N 4.63%) are in good agreement with the theoretical composition (C 32.86%, H 1.95%, N 4.79%) calculated for C_48_H_34_N_6_O_32_Zr_6_ (1754.15 g mol^−1^). In the case of UiO-66-Cys, the presence of sulphur confirmed successful cysteine grafting, and the experimental elemental composition was C 32.6%, H 2.0%, N 5.0%, and S 0.63%. The sulphur content corresponds to ~0.196 mmol of cysteine per gram of MOF and 5.75% functionalisation of the available amine groups. This degree of functionalisation is in good agreement with the value derived from thermogravimetric analysis.

Because surface modification may influence pore accessibility and particle morphology, textural properties and nanoparticle dimensions were subsequently evaluated.

The textural properties of the prepared materials, including pore volume (*V_P_*), BET surface area (*S_BET_*), and pore diameter (*d*), were evaluated from argon adsorption/desorption measurements at −186 °C, and the resulting isotherms are shown in Figure 3D. Both samples exhibit type *I*(b) isotherms according to the IUPAC classification [39,40], which are characteristic of materials possessing predominantly narrow micropores with a small contribution of wider micropores or ultramicropores. The steep uptake at low relative pressures (*p*/*p_0_* < 0.05) confirms the presence of a well-developed microporous network typical for UiO-66-type frameworks, followed by an extended plateau region indicative of the absence of significant mesoporosity. For pristine UiO-66(Zr)-NH_2_, a *S_BET_* surface area of 625 m^2^ g^−1^ was determined, along with a total pore volume *V_P_* of 0.314 cm^3^ g^−1^ and a micropore diameter of approximately 0.64 nm. These values are fully consistent with the expected structural characteristics of the amino-functionalised UiO-66 family [44,45]. Upon post-synthetic modification with Cys, the surface area decreased to 564 m^2^ g^−1^ and the pore volume to 0.275 cm^3^ g^−1^, while the pore diameter increased slightly to 0.68 nm. The mentioned decrease in surface area and pore volume is consistent with the partial occupation of the micropores or pore entrances by Cys molecules grafted to the –NH_2_ groups. The small increase in the calculated pore diameter reflects subtle changes in the local pore environment, likely resulting from alteration of the inner surface chemistry and the replacement of some amino groups by bulkier amide-terminated Cys residues. Importantly, the preservation of the microporous isotherm shape and the absence of hysteresis confirm that the structural integrity of the UiO-66(Zr) framework remains unaffected and no pore collapse or mesostructural alterations occur during functionalisation. Overall, the sorption data corroborate the post-synthetic modification revealed by FTIR, PXRD, TGA and EA analyses, while demonstrating that Cys grafting results in predictable and controlled modulation of the pore accessibility without compromising the robustness of the original UiO-66 architecture. Taken together, the adsorption data, the preserved microporous isotherm shape and the modest decrease in *S_ᴮᴱᵀ_* and pore volume all indicate that Cys is predominantly grafted onto the external surface of the UiO-66(Zr)-NH_2_ crystallites rather than within their internal micropores. The very small reduction in accessible microporosity, combined with the unchanged *I*(b)-type adsorption profile, demonstrates that the pore system remains largely unobstructed. Considering also the relatively large molecular size of Cys compared to the intrinsic pore apertures of UiO-66 and the moderate degree of functionalisation revealed by TGA and EA, the observed structural changes are consistent with surface-localised post-synthetic modification rather than pore filling. This ensures that the framework retains its internal free volume and microporous architecture while acquiring the desired surface functionality for subsequent bioconjugation and targeted cellular interactions.

FIBSEM analysis revealed a predominantly spherical morphology of both samples (Figure 3E,F). The size of the individual nanoparticles within larger agglomerates ranged from 50 to 150 nm, with an average diameter of 82 ± 18 nm for UiO-66(Zr)-NH_2_ (Figure 3E) and from 50 to 200 nm, with an average diameter of 96 ± 28 nm for UiO-66(Zr)-Cys (Figure 3F). The slightly larger average particle size observed for UiO-66(Zr)-Cys can be attributed to partial aggregation induced by Cys surface functionalisation, likely arising from thiol-mediated intermolecular interactions and changes in surface properties.

Beyond structural and morphological validation, molecular-level insight into surface modification and intrinsic optical properties relevant to multimodal imaging was obtained using Raman and fluorescence spectroscopy [46,47].

The Raman spectrum of UiO-66(Zr) particles is shown in Figure 3G (black curve). This spectrum is consistent with previously reported data for the material [48,49,50]. The presence of intense, narrow bands indicates a well-defined, highly ordered crystalline structure, with the bands corresponding to its characteristic molecular vibrations. In particular, the most intense band at 1616 cm^−1^, together with the weak but still well-defined band visible at 1143 cm^−1^ are related to the C=C stretching vibrations of the benzene rings present in the terephthalic acid-moiety. The doublet at 1451 and 1433 cm^−1^ can be assigned to the asymmetric and symmetric *ν*(COO^-^) modes, respectively. Further, the bands at 862 and 633 cm^−1^ are assigned to the out-of-plane CH bending vibrational modes. Finally, a series of very low-intensity bands can also be observed in the skeletal (low-wavenumbers) region. The recorded Raman spectrum confirms that the terephthalate linker remains intact and coordinated to Zr clusters in the framework.

The Raman spectrum of UiO-66(Zr)-NH_2_ particles (Figure 3G, cyan curve) exhibits pronounced changes, indicating substantial structural modifications. The spectrum is dominated by a strong fluorescence background. While the fluorescence background partially limits Raman sensitivity, it simultaneously highlights the suitability of these nanoparticles for fluorescence-based bioimaging. Importantly, the Raman signal remains sufficiently intense and readily detectable. Owing to the high molecular specificity of Raman spectroscopy, together with its capability for microscopic and spatially resolved measurements, this technique, in combination with the investigated nanoparticles, holds considerable promise as part of a multimodal strategy for the detection of selected molecules in targeted applications. In any case, it remains crucial to accurately interpret and assign the individual spectral features and their variations.

The observed spectral modifications are consistent with the aminated analogue of UiO-66(Zr), displaying characteristic UiO-66(Zr) features together with additional contributions from the amino group. Notably, the UiO-66(Zr)-NH_2_ spectrum reveals frequency shifts, particularly of aromatic and carboxylate bands, accompanied by changes in intensity ratios and overall band broadening. Specifically, the band observed at 1616 cm^−1^ shifts to 1625 cm^−1^ and is accompanied by a new band at 1590 cm^−1^, attributed to NH_2_ scissoring/bending vibrations. The doublet at 1280/1256 cm^−1^ corresponds to C–N stretching modes. Further frequency shifts and variations in relative intensity are apparent in the 862/815 cm^−1^ and 828/800 cm^−1^ doublets, reflecting perturbations within the framework. Ultimately, although the 1200–1650 cm^−1^ spectral region is most diagnostic of the influence of the NH_2_ group, the amino substitution also perturbs the low-frequency lattice modes associated with Zr–O vibrations, indicating that the substitution impacts the entire framework.

Raman spectrum of UiO-66(Zr)-Cys particles is also presented (Figure 3G, red curve). Here, the fluorescence background is slightly higher compared to that of UiO-66(Zr)-NH_2_ particles. The spectral characteristics of both functionalised nanoparticles are highly similar, suggesting that Cys primarily binds or influences the MOFs through the -NH_2_ group at identical sites. Beyond this interaction, Cys does not significantly alter the robust framework structure. Given that Cys contains a highly reactive thiol group (-SH), this functionalisation may offer advantages for selective nanoparticle binding to specific targets. However, under the applied experimental conditions (785 nm excitation), the band corresponding to S–H vibrations at approximately 2550 cm^−1^ is essentially undetectable.

The fluorescence properties observed by Raman microspectroscopy were confirmed by fluorescence spectroscopy. Fluorescence emission maxima between 400–475 nm after 280 and 405 nm excitation were observed for both UiO-66(Zr)-NH_2_ and UiO-66(Zr)-Cys nanoparticles (Figure 3H). This fluorescence can be used for fluorescence bioimaging to monitor the distribution of nanoparticles in biological samples [15]. A stronger fluorescence signal was observed for the aminated formulation, while a decrease in the fluorescence of the UiO-66(Zr)-Cys nanoparticles was noted. This may be caused by the presence of Cys, which can create an optical filtering (quenching) effect on the surface of the nanoparticles.

Furthermore, the surface of the nanoparticles was modified with RBD-A488, and the binding of RBD-A488 to UiO-66(Zr)-NH_2_ and UiO-66(Zr)-Cys was confirmed by fluorescence microscopy (an example of nanoparticles conjugated with RBD labelled with A647, shown in red, is presented in Figure S3) and protein separation techniques (sodium dodecyl sulphate polyacrylamide gel electrophoresis of the RBD protein and the residues remaining in the supernatants after RBD conjugation with nanoparticles are shown in Figure S4). Differences in the hydrodynamic diameters of the nanoparticles before and after modification were evaluated using the dynamic light scattering (DLS) method, and the average hydrodynamic diameter (*D_average_*) and polydispersity index (PDI) values are summarised in Table 1. Compared with FIBSEM measurements, larger *D_average_* values of 1700 ± 200 nm were detected for UiO-66(Zr)-NH_2_ in liquid suspension, reflecting the presence of particle agglomerates in solution. This discrepancy can be attributed to the fundamentally different measurement principles, as DLS captures the hydrodynamic size of solvated and dynamically interacting aggregates, which may be influenced by solvent effects and interparticle interactions. Cys modification further increased the *D_average_* values to 4000 ± 100 nm. The increase in hydrodynamic diameter is attributed to thiol-mediated interparticle interactions, leading to partial aggregation in Cys-functionalised nanoparticles. It has been reported that neutral hydrogen bonding and zwitterionic interactions of Cys moieties depend on the local environment and may induce aggregation of gold nanoparticles in solution [51]. Importantly, although DLS yields larger absolute size values than FIBSEM, the observed trend is consistent across both techniques, with Cys-modified UiO-66(Zr) exhibiting larger effective particle sizes and a higher degree of aggregation compared to unmodified UiO-66(Zr)-NH_2_. Interestingly, modification of the particles with RBD-A488 increased the PDI in all studied samples (Table 1), indicating a broader particle size distribution. Nevertheless, the size of UiO-66(Zr)-NH_2_-sm(PEG)_2_-RBD-A488 (2000 ± 100 nm) was only slightly larger than that of the unmodified particles. In contrast, modification with UiO-66(Zr)-Cys-Cys-RBD-A488 and UiO-66(Zr)-Cys-sm(PEG)_2_-RBD-A488 reduced the particle size to 1800 ± 50 nm and 3000 ± 200 nm, respectively. This behaviour can be explained by the reduction in accessible Cys moieties due to the anchoring of SpyCatcher and the SpyTag-functionalised RBD on the UiO-66(Zr)-Cys surface, which likely suppresses thiol-mediated interparticle interactions and thereby limits aggregation.

Taken together, these analyses confirm successful surface functionalisation of UiO-66(Zr)-NH_2_ while preserving crystallinity, porosity, and intrinsic optical properties, providing a suitable platform for subsequent biological investigations.

### 3.2. Targeting of RBD-Modified UiO-66(Zr)-NH_2_ and UiO-66(Zr)-Cys Nanoparticles to Model Cells Overexpressing ACE2 Receptors

The cellular interaction and uptake of RBD functionalised UiO-66(Zr)-NH_2_ nanoparticles were investigated using HEK293T model cells with controlled overexpression of EGFP-tagged ACE2 receptors. Because nanoparticle aggregation can substantially influence cellular association and uptake, targeting experiments were designed with short incubation times to minimise sedimentation-driven effects.

Confocal fluorescence microscopy was first used to assess the intracellular distribution of unmodified UiO-66(Zr)-NH_2_ nanoparticles after 24 h of incubation. In both HEK293T and ACE2r-EGFP-HEK cells, non-specific localisation of the nanoparticles was observed, predominantly in extracellular regions and at elongated distal cellular projections, with no apparent preference for ACE2-expressing cells (Figure S1, Supplementary Material). Confocal images of cells in the supplementary Material also confirm biocompatibility, as cell morphology was not altered at high nanoparticle concentrations. Consistently, flow-cytometric analysis revealed concentration dependent association of UiO-66(Zr)-NH_2_ nanoparticles with cells but no enhanced uptake in ACE2-overexpressing cells compared with non-transfected controls (Figure S2, Supplementary Material). These Supplementary Data quantitatively confirm the absence of ACE2-dependent selectivity for non-functionalised nanoparticles.

The cytocompatibility of UiO-66(Zr)-NH_2_ and UiO-66(Zr)-Cys nanoparticles was subsequently evaluated in HEK293T cells over a concentration range of 0.02–0.07 mg mL^−1^ for 48 h using an MTT assay. No significant reduction in metabolic activity was detected at concentrations below 0.017 mg mL^−1^ for either formulation (Figure S5, Supplementary Material). Quantitative analysis shown in Figure S5 further supports the observed stability of metabolic activity at lower nanoparticle concentrations. At higher nanoparticle concentrations (0.035 and 0.07 mg mL^−1^), an increase in formazan production was observed, which may reflect the catalytic or antioxidant properties of UiO-66(Zr) based materials rather than cytotoxic effects. It should be noted that MTT assay results may be influenced by the redox activity of UiO-66(Zr)-based nanoparticles, which could contribute to increased formazan formation independently of changes in cell viability [15]. These results indicate that both amine- and Cys-modified UiO-66(Zr)-NH_2_ nanoparticles exhibit good biocompatibility under the investigated conditions and are suitable for subsequent targeting studies. Nevertheless, confocal images presented in Figure S1 confirmed that the nanoparticles are highly biocompatible even at high concentrations, in accordance with previous publications [15,52].

Generally, UiO-66(Zr)-NH_2_ material as a drug carrier is well tolerated by different cell types [15,53,54]. However, UiO-66(Zr)-NH_2_ nanoparticles have catalytic properties that can lead to nicotinamide adenine dinucleotide (NAD) plus hydrogen (NADH) regeneration [55]. These mesoporous structures have been shown to co-immobilise dehydrogenase and NAD^+^ for the detection of metabolic markers through the conversion of NAD^+^ to NADH [56]. This could explain the high formazan production at higher concentrations of UiO-66(Zr)-NH_2_ and UiO-66(Zr)-Cys, which may influence oxidation-reduction reactions in cells. It has also been reported that UiO-66(Zr)/UiO-66(Zr)-NH_2_ materials can mimic the action of catalase and peroxidase to decompose highly reactive oxygen species [57,58,59]. This suggests that the biomimetic properties of UiO-66(Zr)-NH_2_ and UiO-66(Zr)-Cys nanoparticles may be active in protecting cells against oxidative stress induced by various factors.

**Figure 4:**
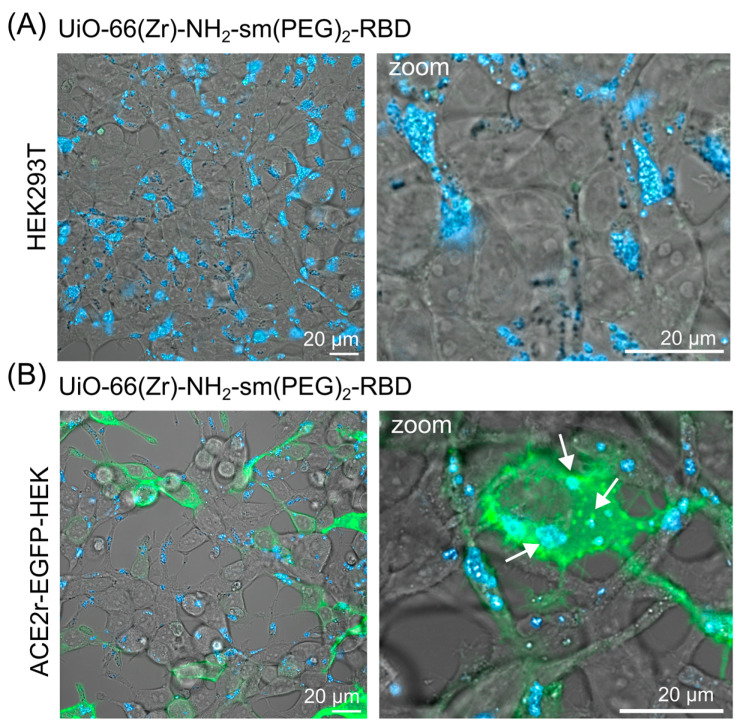
Distribution of 0.07 mg/mL UiO-66(Zr)-NH2 nanoparticles coated with sm(PEG)2 and SARS-CoV-2 RBD-SD1 (UiO-66-NH2-sm(PEG)2-RBD) in (**A**) HEK293T and (**B**) ACE2r-EGFP-HEK cells after 5 h, detected by confocal fluorescence microscopy. Note: In the ACE2r-EGFP-HEK sample, 50% of cells overexpressed ACE2 receptors (green), while 50% of cells showed no ACE2 expression. Thus, both ACE2-positive (green) and ACE2-negative cells are present in the same sample. Zoomed images show the localisation of functionalised nanoparticles within cells. While the specific localisation of the nanoparticles at the place of elongated distal projections was observed in HEK293T cells, small agglomerates of nanoparticles in ACE2r-EGFP-HEK cells can be identified as indicated by the white arrows.

To enable ACE2 receptor-specific interaction, UiO-66(Zr)-NH_2_ and UiO-66(Zr)-Cys nanoparticles were functionalised with the SARS-CoV-2 receptor-binding domain using PEG linker and SpyTag/SpyCatcher bioconjugation. Confocal fluorescence microscopy was performed after a shorter incubation time (5 h) to minimise effects arising from non-specific sedimentation or prolonged exposure. In non-transfected HEK293T cells, RBD-functionalised UiO-66(Zr)-NH_2_-sm(PEG)_2_ nanoparticles were primarily localised at the cell periphery, resembling the distribution of unmodified particles (Figure 4A). In contrast, ACE2r-EGFP-HEK cells displayed distinct intracellular blue-fluorescent nano-agglomerates, frequently confined within vesicular structures indicative of endocytic uptake (Figure 4B, white arrows). These results demonstrate that RBD functionalisation promotes preferential internalisation of the nanoparticles in ACE2 receptor-expressing cells.

To further investigate the influence of surface chemistry on ACE2-mediated uptake, both UiO-66(Zr)-NH_2_ and UiO-66(Zr)-Cys nanoparticles were examined following RBD conjugation and fluorescent labelling of the protein with Alexa Fluor 488 (A488). Aggregation can influence nanoparticle–cell interactions and therefore represents an inherent limitation of the present nanoparticle formulations. Importantly, selective uptake differences between ACE2-positive and ACE2-negative cells were consistently observed under short incubation conditions, supporting a receptor-mediated contribution beyond nonspecific sedimentation effects.

**Figure 5:**
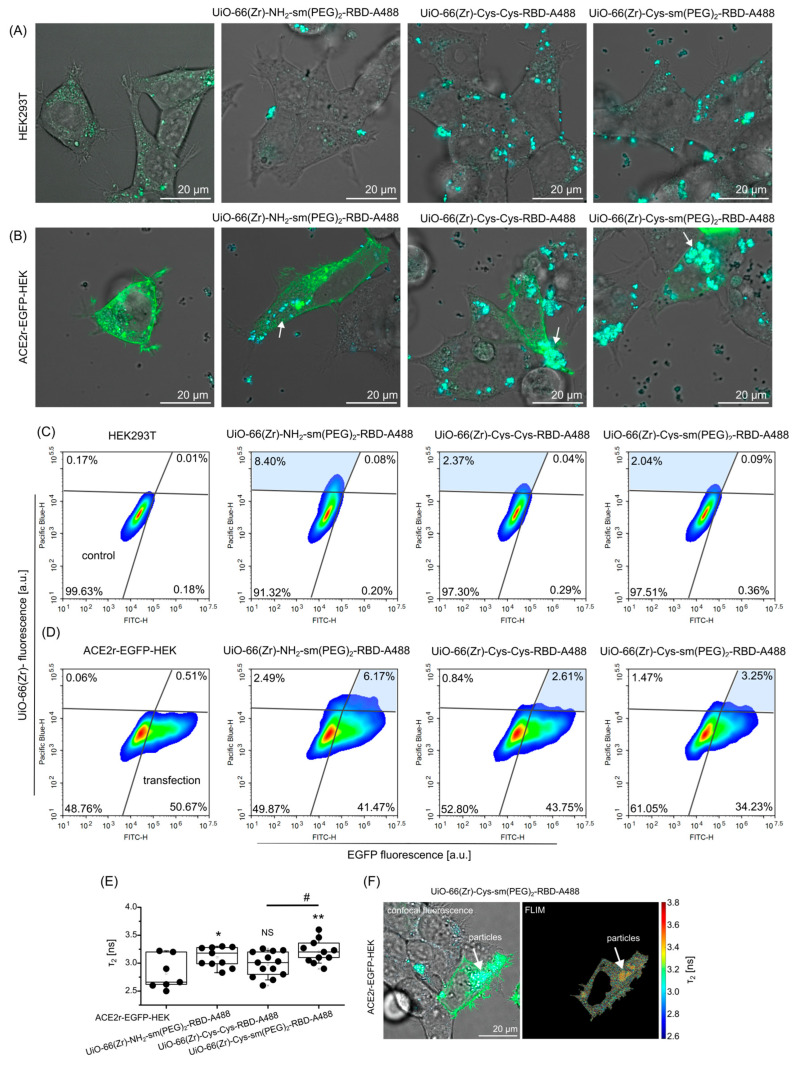
Distribution of 0.009 mg/mL UiO-66(Zr)-NH_2_-sm(PEG)_2_-RBD-A488, 0.01 mg/mL UiO-66-Cys-Cys-RBD-A488, 0.02 mg/mL UiO-66-Cys-sm(PEG)_2_-RBD-A488 nanoparticles (green/blue) in (**A**) HEK293T and (**B**) ACE2r-EGFP-HEK cells (green) incubated for 2 h. Preferential adsorption of the nanoparticles to ACE2r-EGFP-HEK cells is denoted by the white arrow. (**C**) Uptake of these nanoparticles by HEK293T and (**D**) ACE2r-EGFP-HEK cells for 2 h detected by flow cytometry. Light blue regions indicate the populations of high ACE2 receptor-expressing cells with high nanoparticle uptake. The number of events in the density plots is colour-coded from blue to red. (**E**) Statistical analysis of the longer fluorescence lifetime component of EGFP. Level of significant difference from control was determined with a one-way ANOVA test: * *p* < 0.05, ** *p* < 0.01 and NS—not significant, # *p* < 0.05 indicates significant difference between two modifications. (**F**) Illustrative confocal and FLIM images of ACE2r-EGFP-HEK cells incubated with 0.02 mg/mL UiO-66-Cys-sm(PEG)_2_-RBD-A488 nanoparticles for 2 h. Fluorescence lifetimes are colour-coded (blue—short, red—long). The nanoparticles within the cells are marked with a white arrow.

After 2 h incubation, only minimal intracellular fluorescence was detected in HEK293T cells for all tested formulations, indicating low non-specific uptake (Figure 5A). In contrast, co-culture experiments and ACE2r-EGFP-HEK cells revealed a pronounced accumulation of RBD-modified nanoparticles within ACE2 positive cells, often forming intracellular clusters (Figure 5B). Among the tested systems, UiO-66(Zr)-NH_2_-sm(PEG)_2_-RBD-A488 exhibited the highest degree of selective uptake.

These observations were quantitatively confirmed by flow-cytometric analysis. In HEK293T cells, exposure to UiO-66(Zr)-NH_2_-sm(PEG)_2_-RBD resulted in a modest (~8%) increase in the blue-fluorescent population, whereas uptake of the cysteine modified analogues was limited to approximately 2% of the cell population (Figure 5C). In ACE2r-EGFP-HEK cells, uptake of RBD-functionalised nanoparticles was consistently higher in ACE2-positive cells compared with low-ACE2-expressing cells (Figure 5D). Notably, UiO-66(Zr)-NH_2_-sm(PEG)_2_-RBD nanoparticles exhibited approximately two-fold higher uptake than Cys-based formulations, highlighting the influence of surface chemistry and aggregation behaviour on receptor-mediated interactions.

**Figure 6:**
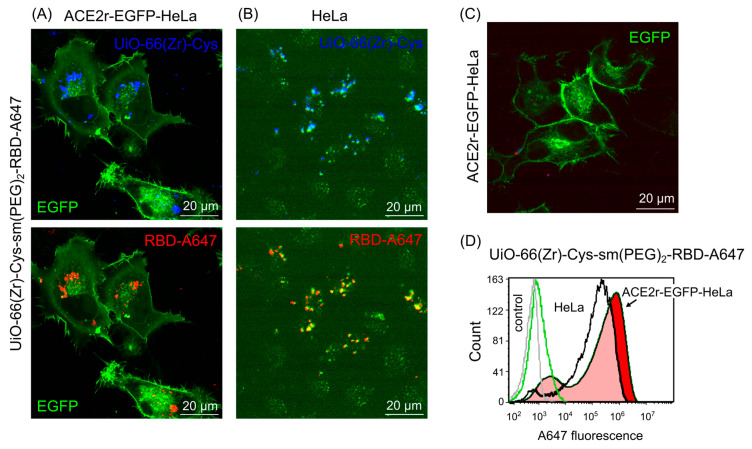
Confocal fluorescence microscopy (3D reconstructions of Z-stacks) images of (**A**) HeLa and (**B**) ACE2r-EGFP-HeLa cells exposed to 0.02 mg/mL UiO-66-Cys-sm(PEG)_2_-RBD-A647 nanoparticles. (**C**) Confocal image of untreated ACE2r-EGFP-HeLa cells. (**D**) Flow cytometric histograms of the red fluorescence intensity indicating the enhanced uptake of UiO-66-Cys-sm(PEG)_2_-RBD-A647 nanoparticles by ACE2r-EGFP-HeLa cells. Control cells (grey: HeLa, green: ACE2r-EGFP-HeLa) were not treated with nanoparticles.

**Figure 7:**
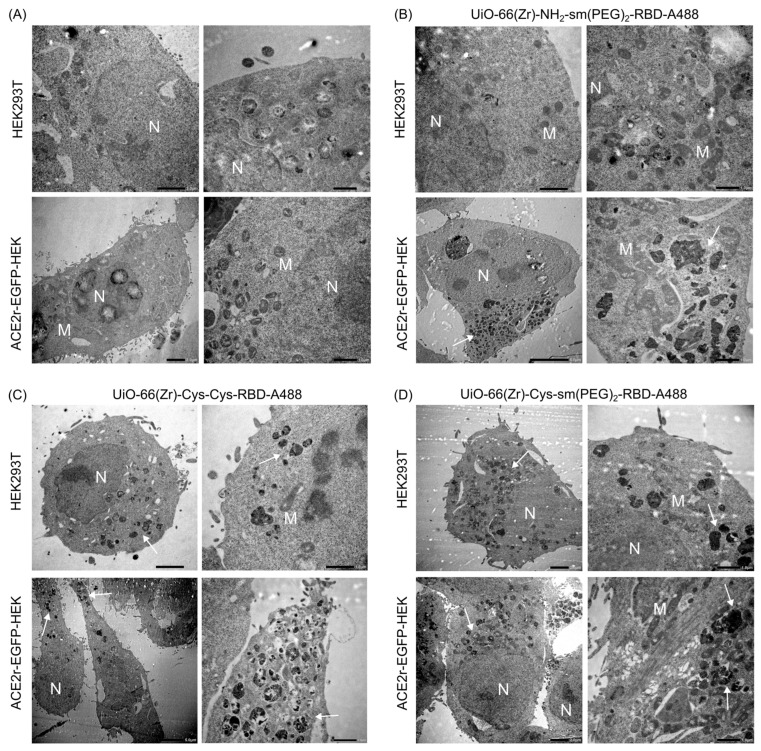
Electron microscopy images of (**A**) HEK293T and ACE2r-EGFP-HEK cells (N-nucleus, M-mitochondria) in the absence and in the presence of (**B**) 0.009 mg/mL UiO-66(Zr)-NH_2_-sm(PEG)_2_-RBD-A488, (**C**) 0.01 mg/mL UiO-66(Zr)-Cys-Cys-RBD-A488 and 0.02 mg/mL UiO-66-Cys-sm(PEG)_2_-RBD-A488 nanoparticles (**D**) nanoparticles (N-nucleus, M-mitochondria, white arrow—endosomal vesicles with nanoparticles).

To further support the internalisation of RBD with modified nanoparticles into ACE2 receptor-expressing cells, HeLa cells (a large and stable cell line suitable for high-resolution microscopy) were also transfected with ACE2r-EGFP. Moreover, to overcome the issue of cellular green autofluorescence and to facilitate determination of the localisation of the RBD, the protein was labelled with AlexaFluor 647 (denoted as RBD-A647). Representative 3D presentations of Z-stack fluorescence images of HeLa and ACE2r-EGFP-HeLa cells (green), both in the absence and presence of UiO-66-Cys-sm(PEG)2-RBD-A647 nanoparticles (red and blue), are shown in Figure 6. This modification was used specifically to demonstrate the internalisation of the modified nanoparticles. ACE2r-EGFP-HeLa cell fluorescence is presented in Figure 6C. While subcellular red fluorescence was detected in all ACE2r-EGFP-HeLa cells (Figure 6A), only some HeLa cells internalised the particles (Figure 6B). Importantly, the blue fluorescence of the nanoparticles and the red fluorescence of RBD-A647 are localised in the same cellular regions. The fluorescence characteristics of the nanoparticles before and after modification are shown in Figure S3 in the Supplementary Material, confirming red fluorescence corresponding to RBD-A647, and thereby verifying successful conjugation of the labelled RBD to the nanoparticles. Higher uptake of UiO-66-Cys-sm(PEG)2-RBD-647 nanoparticles by ACE2r-EGFP-HeLa cells was confirmed by flow cytometry. The histogram of red A647 fluorescence was shifted to the right in ACE2r-EGFP-HeLa cells exposed to UiO-66-Cys-sm(PEG)2-RBD-647 nanoparticles (Figure 7D), suggesting facilitated intracellular transport of the nanoparticles due to targeted modification.

Overall, these results demonstrate that functionalisation of UiO-66(Zr)-NH_2_ nanoparticles with the SARS-CoV-2-RBD confers ACE2 receptor-dependent cellular targeting and internalisation. While Cys-modification provides valuable insight into thiol-mediated interactions and aggregation effects, PEG-linked RBD conjugation to UiO-66(Zr)-NH_2_ yielded the highest selectivity under the investigated conditions. Collectively, the data suggest a receptor-mediated contribution to nanoparticle uptake, although definitive mechanistic confirmation will require receptor-blocking experiments.

To further elucidate whether this preferential uptake reflects a receptor-mediated internalisation pathway, mechanistic studies combining FLIM and electron microscopy were performed.

### 3.3. Mechanistic Insight into ACE2r-Mediated Internalisation Revealed by FLIM and Electron Microscopy

To gain mechanistic insight into the internalisation pathway and intracellular fate of RBD-functionalised UiO-66(Zr)-NH_2_ nanoparticles, FLIM and transmission electron microscopy (TEM) were employed. FLIM provides a sensitive, intensity-independent readout of the local microenvironment [60] and was therefore used to probe receptor-associated interactions and subcellular localisation of nanoparticles in ACE2 receptor-expressing cells.

FLIM analysis of EGFP-tagged ACE2 receptors in ACE2r-EGFP-HEK cells revealed two principal lifetime components (Table S1, Supplementary Material), consistent with previous reports for membrane-associated EGFP [8]. The quantitative parameters provided in Table S1 support the observed trends in fluorescence lifetime behaviour across the analysed conditions. In the presence of RBD-functionalised nanoparticles, a modest but reproducible increase in the longer lifetime component (τ_2_) was observed (Figure 5E). Specifically, τ_2_ increased from 2.8 ± 0.3 ns in untreated cells to 3.1 ± 0.2 ns and 3.2 ± 0.2 ns following incubation with UiO-66(Zr)-NH_2_-sm(PEG)_2_-RBD-A488 and UiO-66(Zr)-Cys-sm(PEG)_2_-RBD-A488 nanoparticles, respectively. No statistically significant change was detected for UiO-66(Zr)-Cys-Cys-RBD-A488, which may reflect partial detachment of RBD-associated with the redox sensitivity of disulfide-mediated coupling.

Spatially resolved FLIM images demonstrated that regions exhibiting prolonged EGFP lifetimes coincided with intracellular blue-fluorescent nanoparticle signals (Figure 5F, white arrows). This spatial correlation is consistent with receptor-associated internalisation and suggests local microenvironmental changes accompanying nanoparticle uptake, although such lifetime changes are not exclusively specific to receptor engagement.

Transmission electron microscopy was subsequently used to visualise nanoparticle localisation at the ultrastructural level. In untreated HEK293T and ACE2-EGFP-HEK cells, typical cellular organelles, including mitochondria and endosomal vesicles, were readily identified (Figure 7A). Following exposure to UiO-66(Zr)-NH_2_-sm(PEG)_2_-RBD-A488, numerous nanoparticle-loaded vesicles with irregular morphology were observed predominantly in ACE2 receptor-expressing cells (Figure 7B). These vesicles likely represent endosome-like and autophagosome-like structures (~1 µm in diameter); however, definitive identification would require marker-based validation.

Cells treated with UiO-66(Zr)-Cys-Cys-RBD-A488 exhibited fewer nanoparticle-containing vesicles overall, with localisation primarily at the cell periphery (Figure 7C). This observation is consistent with partial aggregation and reduced uptake efficiency of Cys-based formulations. In contrast, UiO-66(Zr)-Cys-sm(PEG)_2_-RBD-A488 nanoparticles were detected in vesicular compartments in both HEK293T and ACE2r-EGFP-HEK cells, indicating less pronounced selectivity, likely due to thiol-mediated interactions with membrane proteins independent of ACE2 receptor-expression (Figure 7D).

Taken together, the FLIM and TEM data provide complementary evidence that RBD-functionalised UiO-66(Zr)-NH_2_ nanoparticles are internalised in cells. It is assumed that this internalisation could occur predominantly via an ACE2 receptor-dependent endocytic pathway. These findings further validate the designed nanosystem as a mechanistically well-defined, proof-of-concept platform for receptor-specific biointerface studies.

## 4. Discussion

This study aimed to establish a proof-of-concept MOF-based biointerface platform in which controlled ligand presentation enables preferential interaction with a defined cellular receptor. UiO-66(Zr)-NH_2_ nanoparticles were hierarchically functionalised using cysteine grafting and heterobifunctional PEG linkers to support conjugation of the SARS-CoV-2 receptor-binding domain (RBD) via SpyTag/SpyCatcher chemistry while preserving the crystalline framework and intrinsic optical features relevant for multimodal imaging.

PEGylation of nanoparticles is a well-established approach to improve biocompatibility and drug delivery performance [61]. It was reported that the PEG coating increased the biocompatibility of the unaminated UiO-66 structure used for X-ray computed tomography imaging in vivo [62]. Such modification can enhance nanoparticle stability, enable *pH*-responsiveness, and improve drug delivery to cancer cells [23,63]. However, PEGylation and protein conjugation can also reshape colloidal behaviour in biological media through protein corona formation, and therefore dispersion in PBS may not fully predict behaviour in serum-containing culture medium.

PEG-linked RBD conjugation to UiO-66(Zr)-NH_2_ nanoparticles produced the most pronounced preferential association and uptake in ACE2 receptor-overexpressing model cells across the applied readouts. At the same time, we emphasise that the present evidence remains supportive rather than definitive for ACE2 receptor-mediated targeting because receptor-blocking or competition controls (e.g., excess free RBD, soluble ACE2, or anti-ACE2 antibody pre-treatment) were not included and represent the most direct mechanistic validation.

Cys functionalisation provided insight into how thiol chemistry can increase interparticle interactions and thereby amplify aggregation, which likely contributes to reduced or less consistent apparent selectivity in the cellular assays. Importantly, because RBD loading density was not quantified in the current study, formulation-to-formulation differences in cellular association may reflect not only receptor engagement and ligand accessibility but also differences in effective ligand density on the nanoparticle surface. Quantitative RBD conjugation metrics (e.g., BCA/Bradford of supernatants or calibrated fluorescence of labelled RBD) will be essential in follow-up work to disentangle these contributions.

In addition, MTT-based viability assessment may be influenced by the intrinsic redox activity of UiO-66(Zr)-based nanoparticles, and therefore complementary assays will be required to confirm cytocompatibility independently.

PEGylation may partially mitigate aggregation and nonspecific interactions; nevertheless, micron-scale agglomeration remains a practical constraint. Because aggregation and sedimentation can inflate cell-associated signal, future optimisation should include systematic size/PDI (and ideally ζ-potential) characterisation in water, PBS, and complete culture medium, where protein corona formation can substantially alter dispersion and receptor accessibility.

Including both amine- and Cys-based modification routes is valuable because it demonstrates how subtle surface-chemistry changes can shift colloidal behaviour and cellular association even when the same targeting ligand is used. In this proof-of-concept setting, these comparisons inform design rules for MOF biointerfaces; definitive mechanistic attribution to ACE2 receptor engagement, however, will require the receptor-blocking controls noted above.

Because flow cytometry reports total cell-associated fluorescence (surface-bound and internalised), internalisation conclusions are primarily supported by confocal microscopy (including z-stack approach) and ultrastructural observations, and future work should separate binding from uptake using quenching or acid-wash.

Within this proof-of-concept framework, the observed ACE2-dependent internalisation and endocytic localisation provide a mechanistic context for previously reported virus–receptor interactions. In addition, internalisation of RBD-modified UiO-66(Zr)-NH_2_ and UiO-66(Zr)-Cys nanoparticles into ACE2r-EGFP-HEK cells has been demonstrated, highlighting their potential significance in drug delivery. The use of SpyTag/SpyCatcher chemistry ensured controlled orientation and stable attachment of RBD, while multimodal cellular assays provided functional evidence of receptor-specific interactions. RBD modification of nanoparticles may enable specific targeting and provide a basis for future studies exploring immunogenic or therapeutic responses [64]. While the observed uptake patterns strongly suggest ACE2-mediated interaction, definitive validation through receptor-blocking experiments (e.g., using excess free RBD or anti-ACE2 antibodies) remains an important direction for future work.

Recently, RBD-modified nanoparticles have demonstrated robust and versatile immunogenicity for coronavirus vaccines and have shown potential to protect against SARS-CoV-2 variants [65,66,67]. Furthermore, post-synthetic modification of UiO-66-based MOFs with folic acid, tenofovir, and nystatin has enabled specific interaction with the external spike protein of SARS-CoV-2 [68]. UiO-66(Zr) nanoparticles have also been used in the diagnosis and screening of SARS-CoV-2 with dedicated chips [69]. In the present study, the aim was not to demonstrate antiviral potential but rather to target the same receptors as SARS-CoV-2 to create a reasonable competitor.

Indeed, metal–organic frameworks have shown significant potential for interfering with viral replication processes. Computational studies have demonstrated that MOFs can interact with the SARS-CoV-2 main protease, a key enzyme responsible for viral replication [70]. In addition to this indirect antiviral mechanism, direct interactions between MOFs and the viral spike receptor-binding domain (RBD), including its adsorption on MOF surfaces, were shown in previous study [8]. Beyond direct virus–material interactions, MOFs have also been reported to exert immunomodulatory effects, which may further contribute to antiviral activity by enhancing host immune responses [71,72]. Such immune engagement is considered crucial for effective viral control. Importantly, UiO-66-based nanoparticles have been extensively reported as biocompatible systems suitable for pulmonary drug delivery [21], an application of particular relevance during the COVID-19 pandemic. Taken together, these properties provide a strong rationale for the surface modification of UiO-66-based nanoparticles with SARS-CoV-2 RBD as a targeted and biologically relevant strategy.

Our findings align with reports showing ACE2-mediated endocytosis of RBD-bearing entities, yet uniquely demonstrate that the surface chemistry of the carrier critically modulates this process [73,74]. Modification of UiO-66(Zr)-NH_2_ nanoparticles by the RBD protein enabled entry into ACE2 receptor-overexpressing cells, suggesting that endocytosis and autophagy may contribute to their cellular processing. Moreover, various antiviral agents can be introduced into the cells through the pores of MOFs [14]. To improve treatment efficacy, a promising strategy to neutralise viruses via photodynamic therapy (PDT) can be used [75,76,77]. Photosensitisers, which are active PDT drugs, can be transported to affected cells in MOFs, and their multimodality can be used in diagnostic and therapeutic approaches [8].

The biological experiments (in the present study) were intentionally conducted in controlled ACE2-overexpression models to isolate receptor-associated trends within a proof-of-concept framework. Consequently, these models do not fully represent physiological ACE2 expression, and validation in cells with endogenous ACE2 expression (such as epithelial models) will be an important next step to enhance biological relevance.

While the platform is motivated by virus–receptor recognition and the modularity of MOFs for future cargo loading, this study does not include antiviral efficacy, viral neutralisation, immune-response assays, or therapeutic cargo delivery. Therefore, any broader biomedical applications (including antiviral delivery, immunogenicity, or variant-related considerations) are discussed as future potential rather than demonstrated outcomes.

The primary aim of this study was to establish a receptor-targeted MOF-based biointerface platform rather than to assess antiviral efficacy. Although FLIM and TEM findings are consistent with vesicular internalisation in ACE2 receptor-overexpressing cells, fluorescence lifetime shifts are modest and compartment assignments remain descriptive without marker validation; therefore, mechanistic statements are presented conservatively. Future work will include receptor-blocking controls, quantitative ligand loading, medium-dependent colloidal profiling, and marker-based trafficking assays to enable definitive mechanistic conclusions.

## 5. Conclusions

This study demonstrates the rational surface engineering of UiO-66(Zr)-NH_2_ metal–organic framework nanoparticles to create a virus-mimetic, ACE2-targeted nanosystem suitable for biointerface studies and receptor-specific cellular delivery. Post-synthetic functionalisation with cysteine and polymeric linkers enabled controlled conjugation of the SARS-CoV-2 receptor binding domain while preserving the structural integrity, porosity, and intrinsic optical properties of the MOF platform.

Using model cell lines with controlled ACE2 overexpression, RBD-functionalised nanoparticles exhibited enhanced cellular association and uptake compared with non-targeted controls. Multimodal fluorescence imaging, fluorescence lifetime measurements, flow cytometry, and electron microscopy collectively indicate that cellular internalisation likely proceeds predominantly via ACE2-associated endocytic pathways, with accumulation in endosomal and autophagosomal compartments. Importantly, both amine- and cysteine-modified UiO-66(Zr) nanoparticles showed good biocompatibility within the investigated concentration range, supporting their suitability for biological applications.

While the present work focuses on cellular targeting and biointerface interactions rather than antiviral efficacy, the results establish a versatile proof-of-concept platform that mimics virus–receptor recognition at the nanoscale. The modular architecture of UiO-66(Zr) enables future integration of therapeutic cargoes or imaging agents, as well as adaptation of the targeting ligand to alternative receptors relevant to infectious, inflammatory, or oncological diseases. Taken together, this study highlights MOF-based nanostructures as adaptable tools for receptor-specific biointerface engineering and targeted nanomedical applications.

## Figures and Tables

**Table 1:** Parameters characterising the nanoparticles before and after modifications identified by dynamic light scattering (DLS). Average hydrodynamic diameter—*D_average_*, polydispersity index—PDI. Measurements were performed in triplicate. Significant differences between modifications were determined by one-way ANOVA test: ^###^ *p* < 0.001 (-NH_2_ and -Cys modification), * *p* < 0.05, ** *p* < 0.01, *** *p* < 0.001 (before and after RBD modification), ^NS^-not significant.

	*D_average_* ± SD [nm]	PDI ± SD
**UiO-66(Zr)-NH_2_**	1700 ± 200	0.46 ± 0.01
**UiO-66(Zr)-NH_2_-sm(PEG)_2_-RBD-A488**	2000 ± 100 ^NS^	0.76 ± 0.03 ***
**UiO-66(Zr)-Cys**	4000 ± 100 ^###^	0.43 ± 0.04
**UiO-66-Cys-Cys-RBD-A488**	1800 ± 50 ***	0.72 ± 0.02 **
**UiO-66-Cys-sm(PEG)_2_-RBD-A488**	3000 ± 200 *	0.94 ± 0.07 **

## Data Availability

Data available upon request.

## Supplementary Materials

The following supporting information can be downloaded at: https://www.mdpi.com/article/10.3390/nano16110670/s1, Figure S1: Uptake and subcellular distribution of UiO-66(Zr)-NH2 nanoparticles by HEK293T and ACE2r-EGFP-HEK cells: fluorescence microscopy; Figure S2: Uptake and subcellular distribution of UiO-66(Zr)-NH2 nanoparticles by HEK293T and ACE2r-EGFP-HEK cells: flow-cytometry; Figure S3: Confocal fluorescence microscopy images of UiO-66-Cys-sm(PEG)2-RBD-A647 and UiO-66-Cys-sm(PEG)2-RBD nanoparticles; Figure S4: Sodium dodecyl sulphate polyacrylamide gel electrophoresis of the RBD protein and the residues remaining in the supernatants after RBD conjugation with nanoparticles. Figure S5: Compatibility of UiO-66(Zr)-NH2 nanoparticles with HEK293T cells; Table S1: The parameters derived from fluorescence lifetime analysis of EGFP in ACE2r-EGFP-HEK cells in the absence and presence of RBD-A488-modified nanoparticles determined by FLIM.
