# Supplementary material for: Hierarchical Functionalisation of UiO-66(Zr)-NH2 with Cysteine, PEG, and SARS-CoV-2 Spike RBD to Facilitate ACE2 Receptor Targeting in Model Cells
Source: Nanomaterials (Basel). 2026 May 26;16(11):670. doi: 10.3390/nano16110670 (PMC13258713; doi:10.3390/nano16110670)
Supplement: Supplementary file 1 [file nanomaterials-16-00670-s001.zip › nanomaterials-4303902-supplementary.pdf]

Supplementary Information

Table S1: The parameters derived from fluorescence lifetime analysis of EGFP in ACE2r-EGFP-HEK cells in the absence and presence of RBD-A488-modified nanoparticles determined by time-resolved fluorescence microscopy. The average amplitudes - A_1 and A_2 and corresponding average fluorescence lifetime components τ_1 and τ_2 are shown. The values represent the averages of at least 5 measurements (mean ± S.D.).

EGFP-ACE2r-HEK cells	A_1	A_2	τ_1 [ns]	τ_2 [ns]
-	2 ± 2	5 ± 6	1.8 ± 0.5	2.8 ± 0.3
**UiO-66(Zr)-NH_2-sm$(PEG)_2$-RBD-A488**	4 ± 3	7 ± 6	1.8 ± 0.2	3.1 ± 0.2
**UiO-66-Cys-Cys-RBD-A488**	4 ± 4	5 ± 5	1.6 ± 0.3	3.0 ± 0.2
**UiO-66-Cys-sm$(PEG)_2$-RBD-A488**	5 ± 6	10 ± 11	1.8 ± 0.3	3.2 ± 0.2

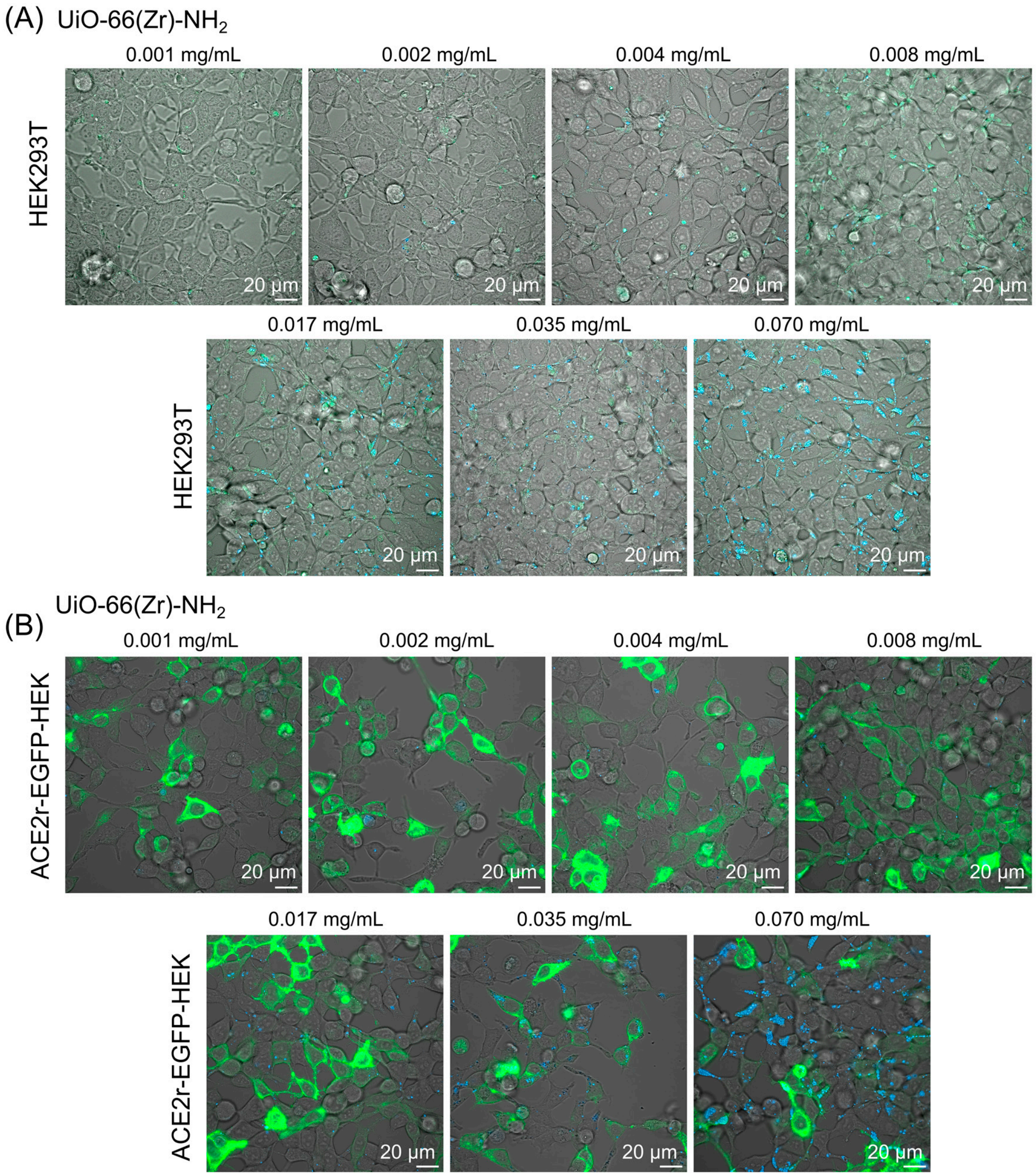

Figure S1: Uptake and subcellular distribution of UiO-66(Zr)-NH_2 nanoparticles by (A) HEK293T and (B) ACE2r-EGFP-HEK cells detected by confocal fluorescence microscopy. Cells were subjected to different concentrations (0.001 – 0.070 mg/mL) of the nanoparticles (blue fluorescence) for 24 hours. The efficiency of the transient transfection of the cells with ACE2r-EGFP is indicated by green fluorescence.

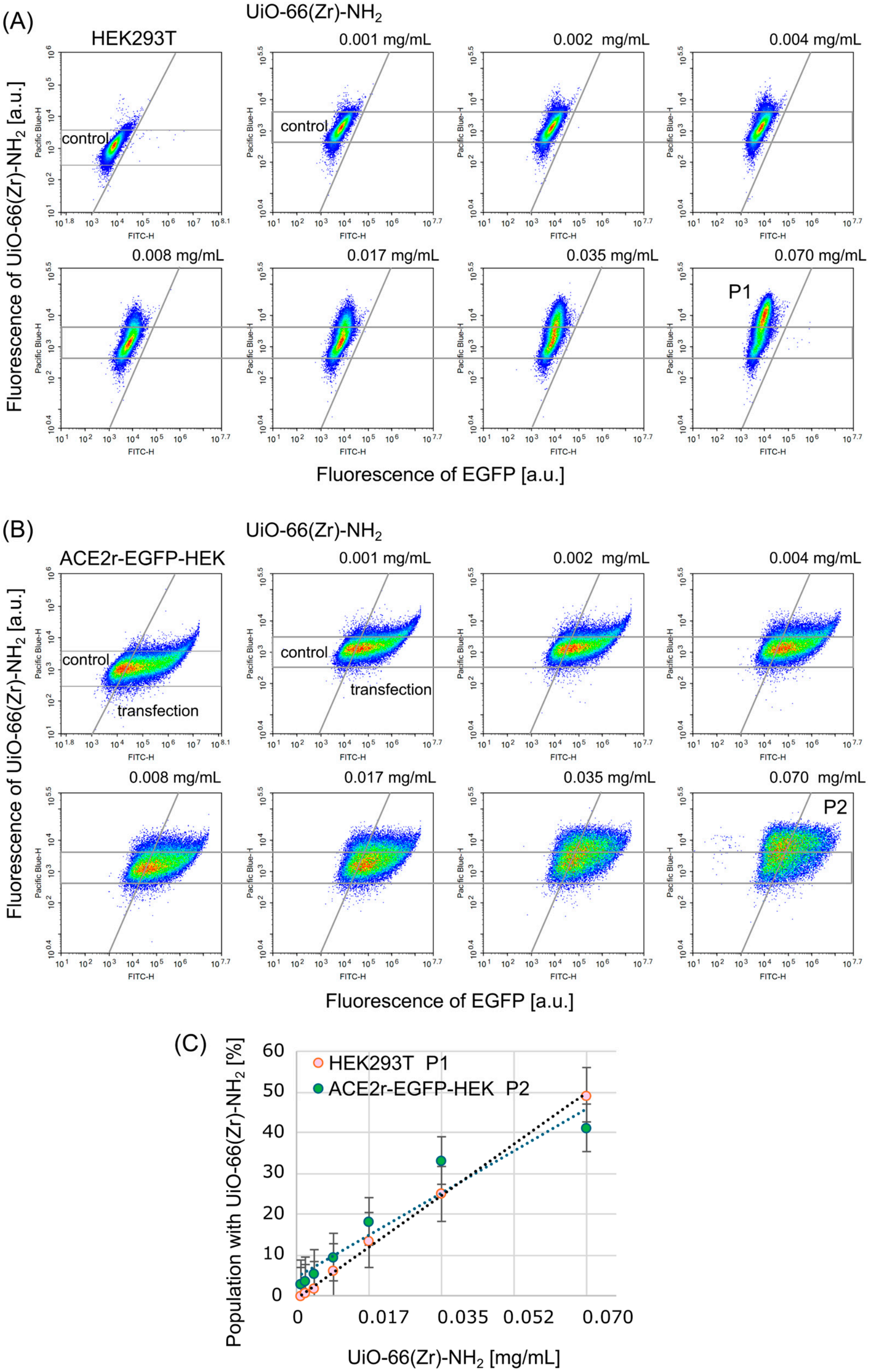

Figure S2: Uptake of UiO-66(Zr)-NH_2 nanoparticles by (A) HEK293T and (B) EGFP-ACE2r-HEK cells. Flow cytometry analysis was performed on cells subjected to different concentrations (0.001 – 0.070 mg/mL) of the nanoparticles (blue fluorescence, gate P1 and P2) for 24 hours. Efficiency of ACE2r-EGFP transient transfection of the cells is indicated by green fluorescence (quadrants on right side of the plots). The number of events detected is colour-coded from blue to red. (C) Concentration dependence of the cell population in P1 and P2, which absorbed UiO-66(Zr)-NH_2 nanoparticles.

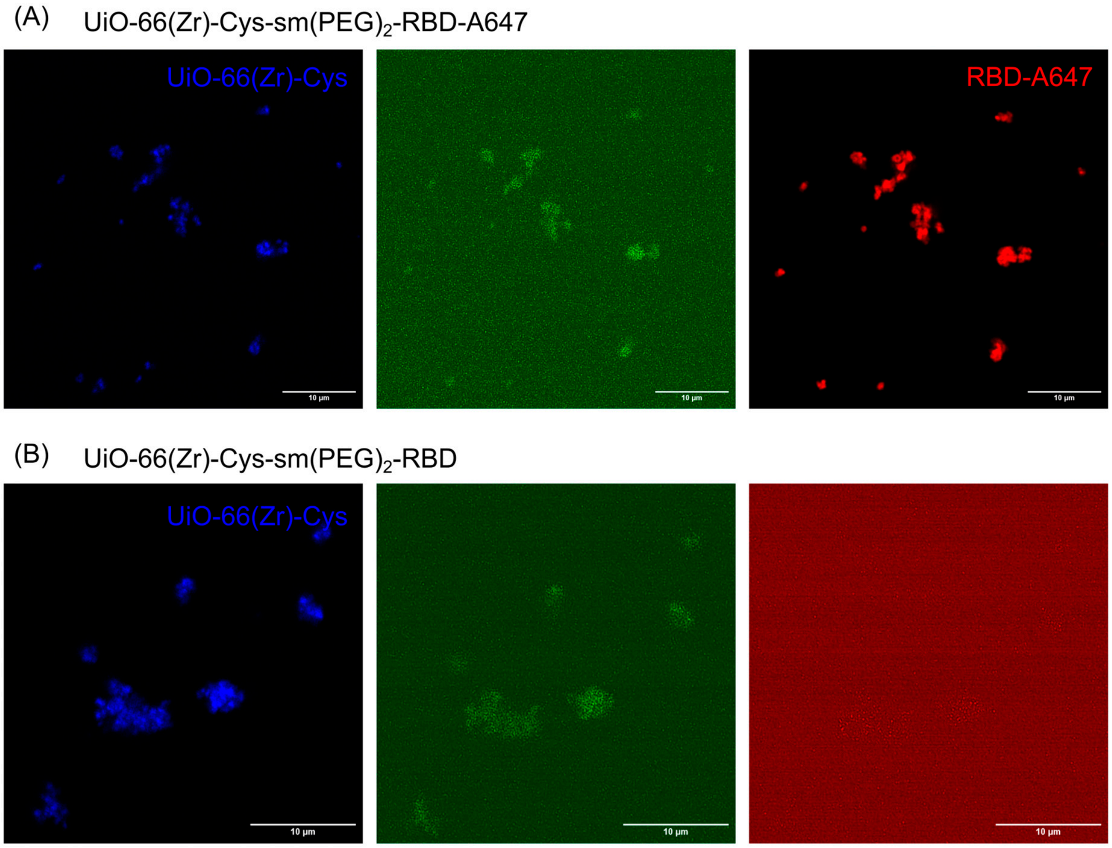

Figure S3: Confocal fluorescence microscopy images of (A) UiO-66-Cys-sm(PEG)$_2$-RBD-A647 and (B) UiO-66-Cys-sm(PEG)$_2$-RBD nanoparticles. Scale bars – 10 µm.

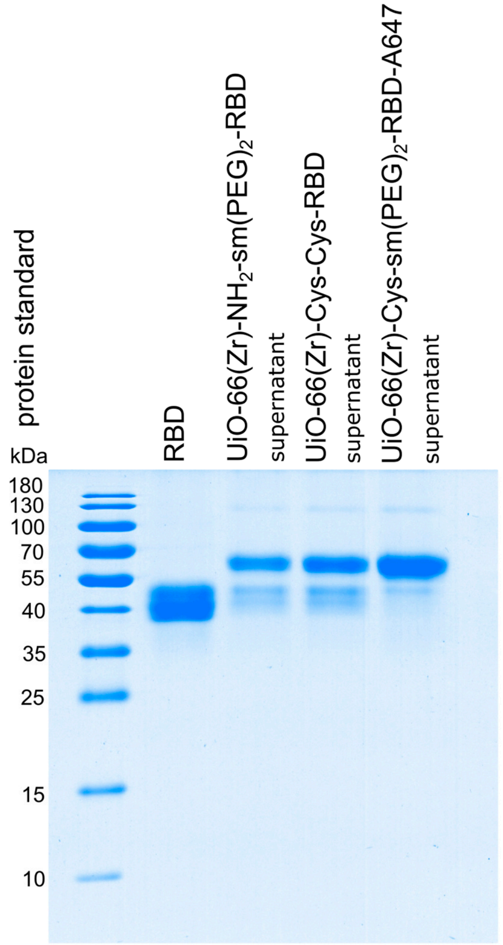

Figure S4: Sodium dodecyl sulfate polyacrylamide gel electrophoresis of the RBD protein and the residues remaining in the supernatants after RBD conjugation with nanoparticles.

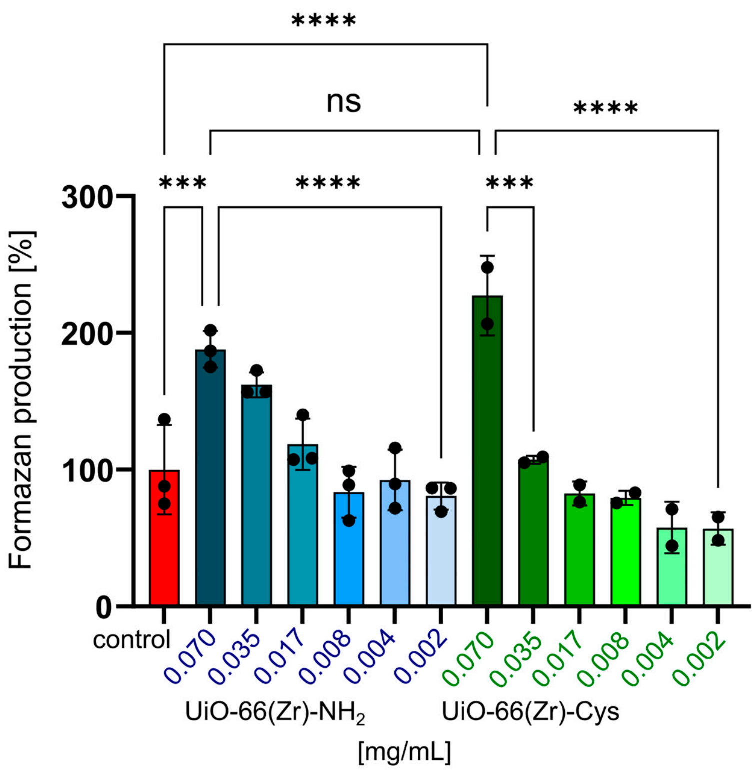

Figure S5: Compatibility of UiO-66(Zr)-NH_2 nanoparticles with HEK293T cells. MTT-assay of cell metabolic activity detected in cells exposed to different concentrations of UiO-66(Zr)-NH_2 (blue columns) and UiO-66(Zr)-Cys (green columns) nanoparticles for 48 hours.
